# Testosterone and pre-androgens by age and menopausal stage at midlife: findings from a cross-sectional study

**DOI:** 10.1016/j.ebiom.2025.105972

**Published:** 2025-10-15

**Authors:** Yuanyuan Wang, Rakibul M. Islam, Molly Bond, Susan R. Davis

**Affiliations:** a Women's Health Research Program, School of Public Health and Preventive Medicine, Monash University, Melbourne, Victoria, 3004, Australia; b Department of Endocrinology and Diabetes, Alfred Health, Melbourne, Victoria, 3004, Australia

**Keywords:** Testosterone, Androgens, DHEA, Menopause, Women

## Abstract

**Background:**

Whether testosterone and the pre-androgens, androstenedione and dehydroepiandrosterone (DHEA) change at menopause remains uncertain.

**Methods:**

The Australian Women's Midlife Years Study recruited a nationally representative sample of 8096 women aged 40–69 years, between 27th October 2023 and 19th March 2024. Participants were excluded from providing a blood sample if pregnant, breastfeeding, using medications that affect sex hormone concentrations, or living over 100 km from a collection centre. Sex steroids were measured by liquid chromatography-tandem mass spectrometry and menopausal status was determined by the Stages of Reproductive Ageing Workshop (STRAW) + 10 criteria.

**Findings:**

Blood samples were provided by 1435 of the 5031 invited participants. After excluding participants with no menopause stage classification, abnormal thyroid function, hyperprolactinemia, bilateral oophorectomy, and an unreported pregnancy, 1104 participants, mean (SD) age 56.5 (8.5) years, were included in the main analysis. Median testosterone concentrations declined between the ages of 40–44 and 55–59 years (median (interdecile range) 0.56 (0.29–1.01) nmol/L vs 0.42 (0.21–0.79) nmol/L, p = 0.001 adjusted for BMI and smoking), reached a nadir at the age of 58–59 years, followed by a modest increase, and did not differ between the youngest group and participants aged 60–64 or 65–69 years. Median androstenedione and DHEA concentrations declined from the age of 40–44 years to 65–69 years, by 51% and 33%, respectively. Testosterone and DHEA concentrations did not vary by menopausal stage in participants aged 48–53 years, whereas androstenedione concentrations were significantly higher in premenopausal, compared with postmenopausal individuals (median (IQR) 1.94 (1.42–2.54) nmol/L vs 1.63 (1.01–2.02) nmol/L, p = 0.001).

**Interpretation:**

Testosterone concentrations declined from the age of 40 years, reaching a nadir at approximately 58–59 years followed by a modest increase, with no impact of natural menopause. These data do not support menopause per se as an indication for testosterone supplementation.

**Funding:**

This research was supported by a 10.13039/501100000925National Health and Medical Research Council (NHMRC) Leadership 3 Investigator Grant award SRD (2016627).

**Research in context**

**Evidence before this study**

Our PubMed search of studies **investigating blood concentrations of testosterone and the pre-androgens, dehydroepiandrosterone (DHEA) and androstenedione, in midlife females revealed past findings were limited by inclusion of individuals using therapies that influence sex steroid production, unreliable ascertainment of menopausal status, use of immunoassays with poor** sensitivity and precision for the measurement of testosterone at low concentrations, **or not adjusting for variables that influence sex steroid blood levels, such as** body mass index and tobacco smoking. Consequently, whether the blood concentrations of these sex steroids change with age or menopausal stage is uncertain.

**Added value of this study**

Our literature search suggests this is the first study to report testosterone and pre-androgen blood concentrations, measured with precision by liquid chromatography-tandem mass spectrometry (LC-MS/MS), in a community-based sample of females aged 40–69 years, not taking medications known to influence endogenous sex steroids, and whose menopausal status was determined by established criteria. Our study shows that blood testosterone concentrations decline until approximately the age of 58–59 years after which concentrations increase with age, while androstenedione and DHEA decline continuously from the age of 40 years. It also reveals no meaningful impact of the natural menopause transition on testosterone concentrations. Additional study strengths include recruitment of a nationally representative sample of non-health-care-seeking participants sufficiently large to allow for examination of the effects of menopause over a narrow age range. Being cross-sectional our findings describe the trend in changes in testosterone and the pre-androgens with age, with reported reference ranges specific to the assay used.

**Implications of all the available evidence**

Our findings, together with those of previous studies, indicate that testosterone concentrations in females aged in their fifties tend to be lower than in younger and older individuals, and that this is a consequence of age, not menopause. Further studies are needed to determine whether the lower testosterone levels at midlife, and the subsequent increase in blood testosterone concentrations in later life, are clinically meaningful.

## Introduction

Testosterone is physiologically important for ovarian function, specifically for growing ovarian follicles and hence for fertility.^1^^,^^2^ In addition, throughout the body testosterone can act directly as an androgen, or indirectly after being converted to oestradiol.^3^ During the reproductive years about half of circulating testosterone is from the ovaries.^4^ An approximately equal amount is synthesised in non-ovarian tissues from the pre-androgens, androstenedione and dehydroepiandrosterone (DHEA), secreted by the ovaries and adrenals, and DHEA after desulphation of DHEA sulphate (DHEAS) from the adrenal glands.^4^ With the loss of ovarian reproductive function at menopause, the adrenal pre-androgens become the main source of testosterone production.^5^

Testosterone blood concentrations, measured with precision by liquid chromatography tandem mass spectrometry (LC-MS/MS), decline in the order of 25% between the ages of 18–39 years in individuals with regular menstrual cycles.^6^^,^^7^ Other studies suggest testosterone blood levels continue to decline through to the 6th decade of life, with a reduced rate of decline during the 6th decade,^8^^,^^9^ followed by an increase from early in the 7th decade of life.^9^^,^^10^ In contrast, DHEA, DHEAS and androstenedione appear to decline continuously with age.^8^^,^^9^^,^^11^^,^^12^ While studies in people aged less than 40 years have measured testosterone with LC-MS/MS^6^^,^^7^ and excluded individuals taking medications that impact testosterone and pre-androgen blood levels,^6^ the studies providing data for females aged 40–69 years either did not comprehensively limit participant medication use,^8^, ^9^, ^10^^,^^12^ or used immunoassays^9^^,^^10^ known to be imprecise at the lowest sex steroid concentrations.^13^^,^^14^ Similarly, while studies suggest testosterone and pre-androgen blood levels do not meaningfully change during the menopause transition,^9^^,^^15^ these studies also have the aforementioned limitations. Despite the remaining uncertainty of female testosterone physiology, claims that testosterone declines at menopause^16^^,^^17^ are fuelling the belief that menopause causes androgen deficiency symptoms which can be alleviated by testosterone supplementation.^18^

Whether blood concentrations of testosterone, DHEA and androstenedione meaningfully vary in females between the ages of 40 and 69 years, or change during the menopause transition, still need to be established. This study was undertaken to determine the associations between age, and menopausal stage, and blood concentrations of testosterone and the pre-androgens, measured by LC-MS/MS in a large, unselected sample of Australian women.

## Methods

### Study design and participants

The Australian Women's Midlife Years (AMY) Study comprised a nationally representative sample of 40–69 year-old individuals recruited between 27th October 2023 and 19th March 2024. The study was designed, and is reported, according to the STROBE guidelines for cross-sectional studies.^19^ As previously described in detail,^20^ geographic representation of our sample was achieved by recruitment according to the latest Australian Bureau of Statistics population distribution of residence of women aged 40–69 years. Recruitment was predominantly by a national probability panel (the Roy Morgan Premium Consumer Panel; 67%) with people on this panel randomly recruited from the general population using random digital dialling to achieve 1000 interviews/week. The remainder (33%) were recruited by Pure Profile who use an internationally accredited research tool that comprises online and offline sources including, but not limited to, internal referral programs, search engine optimisation techniques, location-based registration and radio advertising. Quality checks for the robustness of data collection included IP address matching, name matching, cookies to capture machine identification, exclusion of repeat responders and exclusion of “speeders” (rapid survey completers). Participants were invited to a study that would provide “a comprehensive snapshot of the physical, psychological and socioeconomic wellbeing of Australian women at midlife” with no mention of hormone testing. An explanatory statement was provided and on consent women accessed the online survey. Comprehensive sociodemographic and health data were captured including gynaecological history, medications, and menstrual bleeding details. Weight and height were self-reported. After completing the survey, participants were invited to attend one of 1300 blood sample collection sites across Australia for a blood draw if they had not reported being pregnant, breastfeeding, on an assisted reproduction program, taking systemic sex steroids in the past 4 weeks, glucocorticosteroids, anti-oestrogens, anti-androgens, or gonadotropin-releasing hormone analogues, and did not live more than 100 km from a blood collection centre. If the participant consented, a blood test request form was generated for the participant to print or download. Participants who provided a blood sample received a $50 gift card.

### Assessment of menopausal stage

Menopausal stage was determined using the Stages or Reproductive Aging Workshop (STRAW) + 10 criteria.^21^ In addition to responding affirmatively to “I have not had a period for at least 12 months”, women aged less than 58 years were classified as being postmenopausal if they also did not report having an eating disorder or a body mass index (BMI) less than 18 kg/m^2^.^20^ Vasomotor symptoms (VMS) are not included in STRAW + 10.^21^ Our recent analysis demonstrated that the profiles of people who, according to STRAW + 10, are premenopausal but have changed menstrual flow and VMS are identical to perimenopausal people with VMS and changed cycle length.^22^ This suggests meaningful hormonal changes are occurring in these people before their cycles vary in length. The modified STRAW + 10 classifies women with changed flow and/or cycle length and no VMS as premenopausal and those with VMS as early perimenopausal. Our findings are reported according to both STRAW + 10 and the modified STRAW + 10.^20^

### Sex steroid measurement

Blood samples were drawn within 2 weeks of survey completion, and serum was transported to a central laboratory and stored at −80 °C. Sex steroids were measured in a single serum sample by LC-MS/MS at the ANZAC Research Institute, University of Sydney, Australia as described previously,^6^ with the modifications provided in the Supplementary Information S1. The limits of detection, limits of quantitation, between-day (12 replicates) and within-day (6 replicates) reproducibility (range of coefficient of variation percentage for three quality control levels) for each analyte were as follows: testosterone, 0.035 and 0.087 nmol/L, 2%–9%, and 3%–7%; androstenedione, 0.087 and 0.17 nmol/L, 4%–8%, and 3%–6%; and DHEA, 0.069 and 0.17 nmol/L, 3%–5%, and 8%–12%, respectively. For all analytes, the external recovery was 85%–110%, and the matrix effect was 85%–93%. Sex hormone binding globulin (SHBG) was measured by Siemens Centaur Atellica (Siemens Healthcare Ltd, Hawthorn East, Vic., Australia 3123) with CVs ranging from 3.4 to 4.1% on samples with SHBG concentrations between 9.62 and 52.15 nmol/L.

### Participants sample size

The AMY Study was powered to estimate the association between moderate to severe VMS and poor work ability. The final number of participants in the present study was determined by the number of women invited to provide a blood sample who then attended a sample collection centre. With 1104 participants, we were more than adequately powered to perform linear regression analysis with 4 variables in each of the models.^23^

### Ethics

The study was approved by the Monash University Human Research Ethics Committee (ID 386643). Participation required provision of online informed consent.

### Statistical analysis

Descriptive statistics are reported using mean with standard deviation (SD), or median with interquartile range (IQR) or 10th and 90th percentiles (interdecile range; IDR) for continuous variables, and number with percentage for categorical variables, where appropriate. Due to the positively skewed distribution of sex steroid concentrations, Generalised Linear Models with a Gamma distribution and a log link were used to examine the associations of BMI and smoking with sex steroid concentrations. Concentrations of testosterone, DHEA, and androstenedione are reported by 5-year age groups as median, range, and IDR. The associations between age and sex steroid concentrations are presented graphically as scatter plots with locally weighted scatterplot smoothing (Lowess) curves and box and whisker plots. To explore the association between age and sex steroid concentrations, curve estimation techniques were applied, and a quadratic term for age (age^2^) was included in the linear regression models to model the nonlinear relationships. The associations between menopausal stage and sex steroid concentrations were explored in women aged 48–53 years, as this was the age range in which there was the most balanced distribution of premenopausal, perimenopausal and postmenopausal participants for comparison. For each sex steroid, the log-transformed steroid concentrations were further compared based on age groups and menopausal stage, adjusted for age, BMI and smoking where appropriate, using the general linear F-test with Bonferroni post-hoc analyses. Additional analyses were performed comparing the sex steroid concentrations between postmenopausal women who reported bilateral oophorectomy and postmenopausal women with at least one ovary. A p value < 0.05 (two-sided) was considered statistically significant. No outliers were excluded from any analysis. Analyses were performed using Stata MP (version 16.0; StataCorp LP, College Station, Texas) and IBM SPSS Statistics (version 27.0.0.0; IBM Corp., NY).

### Role of the funding source

The funder (NHMRC) had no role in the study's design, conduct, and reporting. All authors had complete access to all the study data and take final responsibility for the decision to submit for publication.

## Results

**Fig. 1:**
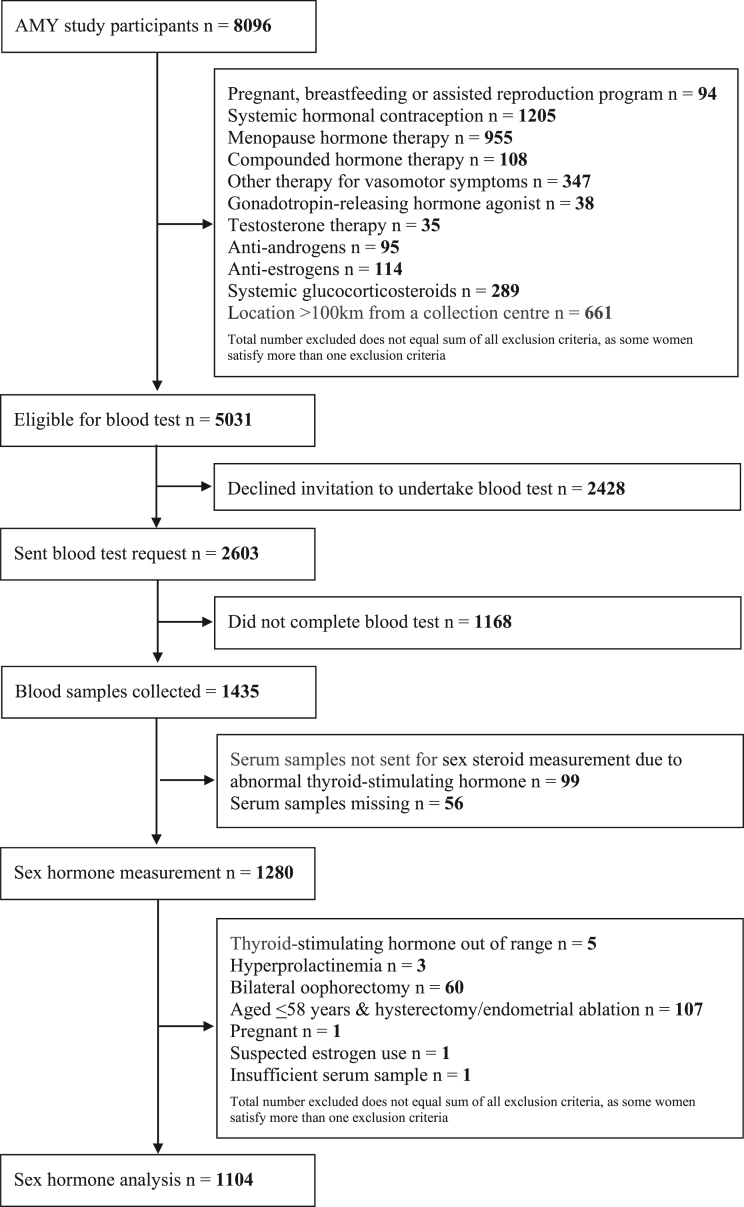
Inclusion of participants for sex steroid analysis.

After excluding the 3065 of the 8096 Amy Study participants according to the prespecified criteria, 5031 participants were invited to provide a blood sample. Of these, 2428 declined, 1168 accepted but did not attend for a blood draw, and 1435 provided a blood sample of which 1432 serum samples were received at the central laboratory. The 1280 samples with thyroid stimulating hormone (TSH) in the normal range were sent for sex steroid measurement, with one sample being insufficient for measurement. We excluded from the main analysis 60 participants who reported bilateral oophorectomy, 107 aged ≤58 years who could not be classified by STRAW + 10 (amenorrhoea due to prior hysterectomy, endometrial ablation, or a progestin intrauterine device), three with hyperprolactinaemia, a further five with their TSH out of range, one found to be pregnant, and one with suspected oestrogen use, leaving 1104 participants for sex steroid analysis (Fig. 1).

We have previously demonstrated that the 5509 AMY Study participants classified by STRAW + 10 were similar to Australian women of the same age by multiple characteristics, and by geographic distribution, according to the latest census data.^20^ As shown in Table 1, the characteristics of the 1104 women included in the present analysis were similar to the 4405 AMY Study participants classified by STRAW + 10 who either did not provide a blood sample, or were excluded from the sex steroid analysis. Most included in the analysis were of European ancestry (n = 974, 88%). Their mean age was 56.5 (SD 8.5) years, their mean BMI was 29.0 (SD 7.2) kg/m^2^, with 29% (n = 319) overweight, 39% (n = 426) having obesity, and 9% (n = 101) current tobacco smokers. Overall, 243 (22%) were premenopausal, 122 (11%) perimenopausal, and 739 (67%) postmenopausal.

**Table 1:** Characteristics of the study participants, and participants able to be classified by STRAW + 10 who did not have blood draw or were excluded.

	Participants included in the analysis n = 1104	Participants able to be classified by STRAW + 10 who did not have blood draw or were excluded n = 4405
Age (years), mean (SD)	56.5 (8.5)	55.8 (8.8)
Age group, n (%)		
40–44	128 (11.6)	646 (14.7)
45–49	147 (13.3)	621 (14.1)
50–54	167 (15.1)	599 (13.6)
55–59	188 (17.0)	773 (17.5)
60–64	241 (21.8)	849 (19.3)
65–69	233 (21.1)	917 (20.8)
Ancestry		
European ancestry	974 (88.2)	3735 (84.8)
Aboriginal or Torres Strait Islander	34 (3.1)	244 (5.5)
Asian	60 (5.4)	274 (6.2)
Other	36 (3.3)	152 (3.5)
BMI (kg/m^2^), mean (SD)^a^	29.0 (7.2)	29.4 (7.6)
BMI (kg/m^2^) categories, n (%)^a^		
<18.5	19 (1.7)	95 (2.2)
18.5–<25	337 (30.6)	1288 (29.3)
25–<30	319 (29.0)	1266 (28.8)
30–<40	343 (31.2)	1329 (30.3)
≥40	83 (7.5)	414 (9.4)
Tobacco smoker, n (%)		
Never/past	1003 (90.9)	3808 (86.4)
Current	101 (9.1)	597 (13.6)
Alcohol consumption in the past month, n (%)		
No	373 (33.8)	1754 (39.8)
Yes	731 (66.2)	2651 (60.2)
Menopausal status, n (%)		
Premenopausal	243 (22.0)	1007 (22.9)
Perimenopausal	122 (11.1)	493 (11.2)
Postmenopausal aged <55 years	89 (8.1)	360 (8.2)
Postmenopausal aged ≥55 years	650 (58.9)	2222 (50.4)
Surgical postmenopausal	0	323 (7.3)

Data presented as mean (SD) or n (%).

a n = 1101 and n = 4392, respectively.

With sex steroid concentrations as the dependent variable in the Generalised Linear Models with a log link, current smoking was associated with a 14.3% higher concentration of testosterone (β-coefficient 0.134 nmol/L, p = 0.046) and 24.4% higher concentration of androstenedione (β-coefficient 0.218 nmol/L, p < 0.001), adjusted for age and BMI. Each unit increase in BMI was associated with testosterone concentrations being 0.7% greater (β-coefficient 0.007 nmol/L, p = 0.01) and DHEA concentrations 0.6% less (β-coefficient −0.006 nmol/L, p = 0.003), adjusted for age and smoking (Supplementary Table S1).

**Fig. 2:**
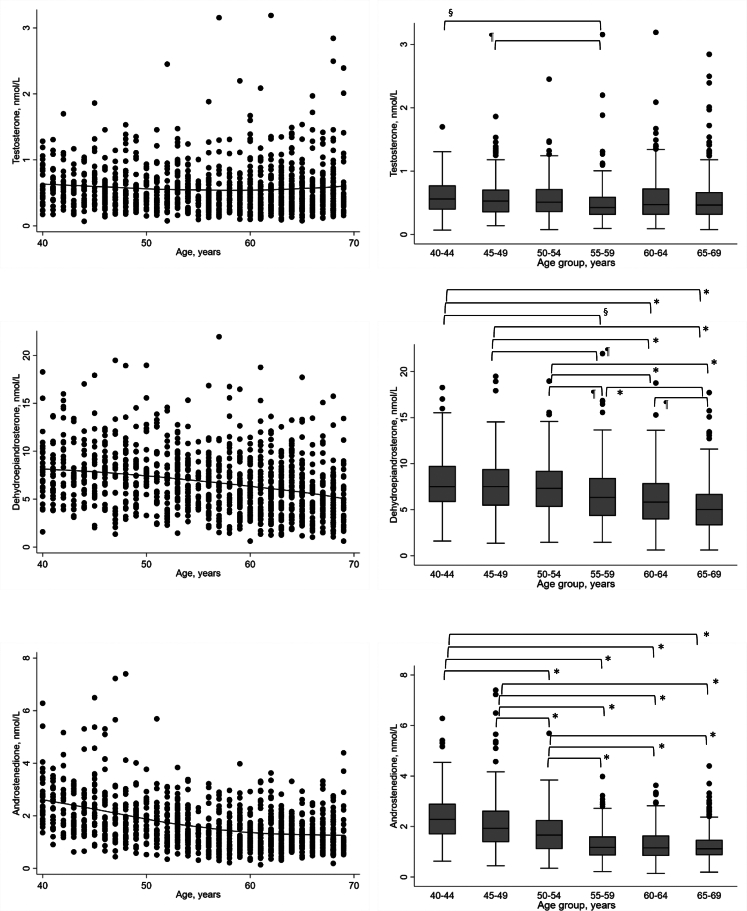
Association between age and sex steroid concentrations. Footnote: p value for between-group difference adjusted for body mass index and smoking using the general linear F-test with Bonferroni correction (only p values for statistically significant differences were shown). ∗p < 0.001, ^§^p = 0.001, ^¶^p < 0.05. Lowess curves are shown as solid lines in the left panel. The boxes show the median values and interquartile ranges, and the whiskers represent 1.5 times the interquartile range from the box.

Median testosterone concentrations exhibited a 25% decline between the ages of 40–44 and 55–59 years (median (IDR) 0.56 (0.29–1.01) nmol/L vs 0.42 (0.21–0.79) nmol/L, p = 0.002, general linear F-test), which remained significant after adjustment for BMI and smoking, p = 0.001 (Table 2). There was no significant difference in SHBG between these two age groups (54.0 (31.0–100.0) nmol/L vs 51.0 (25.0–98.0) nmol/L) (Supplementary Table S2). The Lowess curve for testosterone revealed a nonlinear change with age (Fig. 2) and accordingly, testosterone concentrations were not statistically different between the youngest and two oldest groups. When a quadratic term (age)^2^ was added to the regression model, together with BMI and smoking, the model predicted a nadir in testosterone at 58–59 years of age, with a slight increase thereafter. The model explained 2.6% of the variation in testosterone concentrations (Supplementary Table S3). Median androstenedione and DHEA concentrations declined from the age of 40–44 years to 65–69 years, by 51% and 33%, respectively. The model for androstenedione predicted a continuous decline over the full age range with a diminished rate of decline from 66 to 67 years of age and explained 22.6% of the variation in androstenedione. The β-coefficients for DHEA were negative in both the linear and quadratic regression models indicating a linear association between age and DHEA, with the lowest concentrations by the age of 65–69 years, with no meaningful difference in the variation in DHEA explained by the two models (explaining 10.4% and 10.7%, respectively).

**Table 2:** Sex steroid concentrations by age group.

	Age group (years)					
	40–44	45–49	50–54	55–59	60–64	65–69
n	128	147	167	188	241	233
**Testosterone (nmol/L)**						
Mean	0.61	0.59	0.56	0.49	0.56	0.57
Median	0.56^a^	0.53^b^	0.51	0.42^a,b^	0.47	0.46
Standard deviation	0.29	0.32	0.31	0.34	0.38	0.43
Minimum	0.07	0.14	0.08	0.10	0.09	0.08
10th percentile	0.29	0.26	0.26	0.21	0.20	0.22
90th percentile	1.01	1.11	0.95	0.79	1.01	1.04
Maximum	1.70	1.86	2.45	3.15	3.19	2.84
**Dehydroepiandrosterone (nmol/L)**						
Mean	7.98	7.62	7.52	6.59	6.12	5.43
Median	7.51^c,d,e^	7.52^f,g,h^	7.32^i,j,k^	6.34^c,f,i,l^	5.82^d,g,j,m^	5.03^e,h,k,l,m^
Standard deviation	3.16	3.20	3.11	3.13	2.91	2.83
Minimum	1.59	1.35	1.46	1.46	0.62	0.62
10th percentile	4.26	3.85	3.74	3.12	2.77	2.29
90th percentile	12.10	11.37	12.10	9.92	10.05	8.74
Maximum	18.27	19.48	18.96	21.95	18.76	17.72
**Androstenedione (nmol/L)**						
Mean	2.40	2.18	1.72	1.32	1.31	1.27
Median	2.28^n,o,p,q^	1.93^r,s,t,u^	1.66^n,r,v,w,x^	1.17^o,s,v^	1.15^p,t,w^	1.12^q,u,x^
Standard deviation	0.97	1.21	0.79	0.66	0.64	0.63
Minimum	0.63	0.44	0.35	0.22	0.14	0.19
10th percentile	1.35	1.03	0.84	0.60	0.67	0.63
90th percentile	3.46	3.56	2.68	2.27	2.13	2.13
Maximum	6.28	7.40	5.69	3.98	3.63	4.40

^d,e,g,h,j,k,l,n,o,p,q,r,s,t,u,v,w,x^p < 0.001, ^a,c^p = 0.001, ^b^p = 0.02, ^f^p = 0.03, ^i^p = 0.04, ^m^p = 0.046, between-group differences adjusted for body mass index and smoking with Bonferroni correction.

**Fig. 3:**
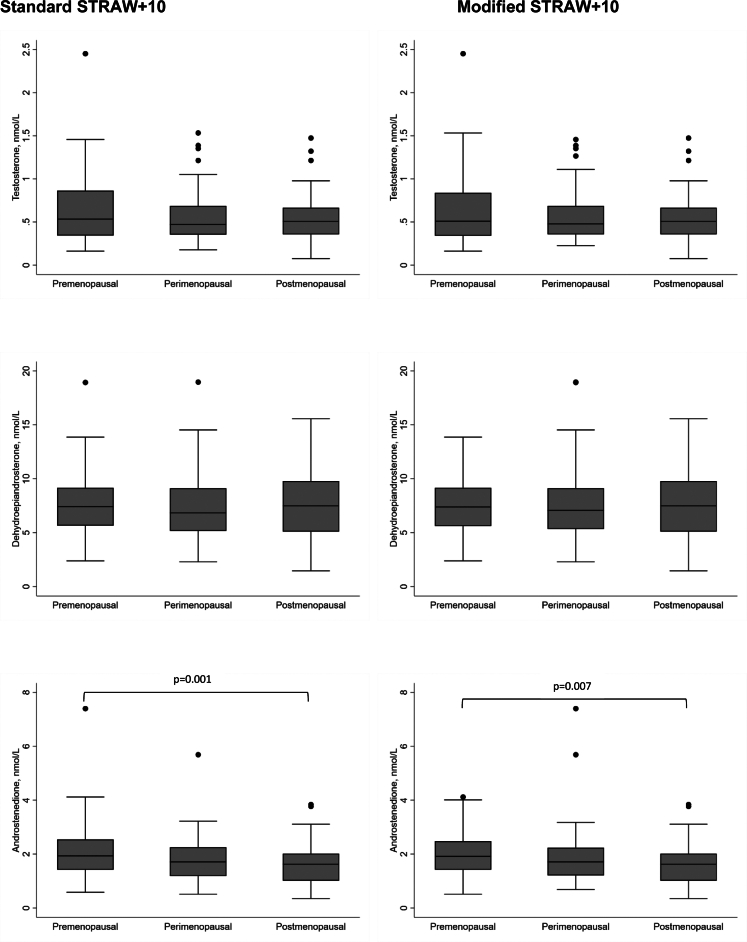
Association between menopausal stage and sex steroid concentrations in women aged 48–53 years. Footnote: p value for between-group difference adjusted for age, body mass index, and smoking using the general linear F-test with Bonferroni correction (only p values for statistically significant differences were shown).

The associations between menopausal stage and the sex steroids were examined using STRAW + 10 and the modified STRAW + 10 criteria in 192 women aged 48–53 years, with adjustment for age, BMI and smoking (Fig. 3; Supplementary Table S4). Testosterone and DHEA did not vary between menopausal stages applying either STRAW + 10 criteria. Androstenedione concentrations were significantly higher in premenopausal, compared with postmenopausal individuals (median (IQR) 1.94 (1.42–2.54) nmol/L vs 1.63 (1.01–2.02) nmol/L, p = 0.001, general linear F-test) using the STRAW + 10 criteria, which remained significantly different between premenopausal and postmenopausal individuals using the modified STRAW + 10.

Testosterone concentrations were significantly lower in the 60 postmenopausal women who reported bilateral oophorectomy compared with postmenopausal women with at least one ovary, adjusted for age, BMI and smoking (median (IQR) 0.33 (0.25–0.49) nmol/L vs 0.45 (0.32–0.66) nmol/L, p < 0.001, general linear F-test), whereas DHEA and androstenedione concentrations did not differ significantly between the two groups (Supplementary Table S5).

## Discussion

In this cross-sectional study, designed to expand our knowledge of the physiology of testosterone and the pre-androgens in midlife females, testosterone blood concentrations diminished from the age of 40 years through to the end of the 6th decade, and from that time modestly increased. While DHEA declined linearly with age, the rate of decline in androstenedione lessened as age increased. The only steroid to decline in relation to menopause was androstenedione.

Our previous community-based study of 595 females, aged 18–75 years, showed a 65% decline in testosterone measured by immunoassay between the ages of 18 and 62 years, with the nadir at 60–62 years followed by a small increase, as seen in the present study.^9^ We subsequently reported sex steroid concentrations, measured by LC-MS/MS, in females aged 18–39 years^6^ and 70–94 years,^11^ with no identifiable factors that might influence their sex steroids.^11^ We found testosterone concentrations declined by 25% between the ages of 18–39 years,^6^ which was less than previously reported with immunoassays.^9^^,^^24^ The present study demonstrates blood testosterone concentrations continue to decline by a further 25% from the age of 40 years through to 58–59 years, after which testosterone gradually increases. These changes do not appear to be explained by changes in SHBG concentrations which did not parallel the changes seen in testosterone concentrations. The lack of a meaningful effect of age across midlife on SHBG concentrations seen in this study was also seen in our prior, large community-based study.^9^ This suggests the increase in testosterone concentrations we previously identified in females from the age of 70 years, in both cross-sectional^11^ and longitudinal^25^ analyses, commences about a decade earlier. A consistent finding across all studies to date has been a continuous decline in DHEA and androstenedione with age from the end of the second decade,^6^^,^^8^^,^^9^^,^^11^ discrepant to the small, but progressive increase in testosterone from around the age of 60 years.^9^^,^^11^ Circulating levels of DHEA are several fold greater than testosterone throughout the adult female lifespan, indicating that biosynthesis of testosterone is dependent on enzyme activity, not precursor availability.

Testosterone and DHEA concentrations were unaffected by menopausal status, as previously reported in studies that used immunoassays.^9^^,^^15^ The menopause-associated fall in androstenedione was not unexpected as functioning ovaries contribute over 50% of androstenedione to the circulation, whereas the estimated ovarian contribution of DHEA is in the order of 10–20%.^26^ The higher blood testosterone concentrations in smokers and people who were overweight or had obesity,^9^^,^^10^ and the inverse association between DHEA concentrations and BMI, while not new,^27^^,^^28^ demonstrate the importance of adjusting for these variables. Also as previously reported,^9^ bilateral oophorectomy was associated with lower testosterone concentrations in postmenopausal women, irrespective of age.

Our findings have several clinical implications. We have observed that in early perimenopause the prevalences of both sexual desire and arousal dysfunction are double those of premenopausal females.^29^ Despite this, no meaningful change in testosterone was seen in relation to the menopause transition in the present analysis. Therefore together, these findings do not support testosterone supplementation as a physiological replacement therapy in relation to the menopause transition. However, the nadir in testosterone around the age of 58–59 years corresponds to the peak prevalence of low sexual desire, arousal, and sexual self-image observed in the AMY Study sample.^29^ This fits with testosterone as a pharmacotherapy consistently being found to improve sexual desire and arousal in naturally and surgically postmenopausal women who have low sexual desire causing them distress.^30^^,^^31^ Conversely, systematic reviews of observational studies^32^, ^33^, ^34^ and randomised placebo-controlled trials^35^ have not shown testosterone supplementation confers other health benefits, such as prevention of muscle loss, cognitive benefits or improved mood, or increased bone density, with the caveat that many studies reporting these outcomes had major design limitations. Therefore, our finding of a nadir in testosterone around the age of 58–59 years should not be interpreted as justification for testosterone treatment of females of this age for these other purposes.

The gradual increase in testosterone from the end of the 6th decade, in parallel with declining concentrations of the precursor steroids, DHEA and androstenedione,^10^^,^^11^^,^^25^ may be of physiological significance. In the Sex Hormones in Older Women Study, participants aged 70 years and older with testosterone concentrations in the lowest 25th centile had a more adverse lipid profile,^36^ plus a 2-fold greater risk of a major ischaemic cardiovascular event over a 4.4 year follow up.^37^ Participants with higher testosterone concentrations also had less decline in hand grip strength,^38^ and were less likely to have moderate to severe depressive symptoms.^39^ These findings are not evidence that testosterone therapy is beneficial for older women. Whether these findings reflect direct testosterone effects or testosterone serving as a surrogate marker for better health needs investigation.

As our study was cross-sectional it cannot be assumed that the changes observed will occur in any given individual with age. Verification of our findings in a longitudinal study would be ideal. This would require recruitment of a larger sample to ensure retention of sufficient participants who do not commence therapies, or have surgical interventions, that would affect their endogenous sex steroids during follow-up. As our study population was predominantly of European ancestry the findings may not be generalisable to other populations. Additionally, as LC-MS/MS assays are not harmonised, the absolute values reported in our study should not be used as reference ranges for other assays, but instead provide a guide to the changes one would anticipate using other validated methods. Further studies are needed to determine whether absolute values of testosterone and the pre-androgens have meaningful clinical correlations in midlife women.

Study strengths include limiting our analysis to participants free of factors modulating their endogenous sex steroid blood levels with adjustments for age, BMI and smoking in order to explore female physiology. We did not exclude, or categorise, participants based on past reproductive history as our goal was to capture the full range of testosterone and pre-androgen concentrations in people not on therapy that would modulate their sex hormones in the general community. Furthermore, sex steroids were measured by LC-MS/MS, which provides precision at testosterone concentrations below the limit of detection of most immunoassays, and has the added advantage of accurately measuring several steroids simultaneously in a single sample.^40^ To examine the impact of the menopause transition we only included participants whose menopausal status could be classified by STRAW + 10 criteria and limited this analysis to the years in which there were similar numbers of individuals who were premenopausal, perimenopausal and postmenopausal. Furthermore, we examined the effect of menopause using both STRAW + 10 and the modified STRAW + 10 criteria in order not to miss a nuanced impact of early perimenopause heralded by the onset of VMS.

In summary, our findings, together with our more recent studies in younger and older females, provide a model of the physiological variations in testosterone and the pre-androgen concentrations across the female adult lifespan. This model indicates that future studies of potential beneficial effects of testosterone therapy be age specific, rather than simply postmenopausal people as a whole.

## Contributors

SRD originally conceptualised the AMY Study, SRD and MB developed the methods, supervised data collection, and maintained oversight of the project during data collection. SRD and MB oversaw data collection and MB, RMI and YW conducted data cleaning. All authors were permitted to access the raw data. SRD and YW accessed and verified the underlying data. YW conducted all analyses for the manuscript and SRD reviewed all analyses. SRD wrote the original draft of the manuscript which was reviewed by YW and refined by all authors. All authors read and approved the final manuscript and accept responsibility to submit for publication.

## Data sharing statement

The AMY dataset belongs to the Women's Health Research Program, Monash University. Currently, the dataset is subject to further analyses. However collaborative work would be considered on request to the study authors following an established protocol.

## Declaration of interests

SRD has prepared and delivered educational presentations for Health Ed Australia has served on Advisory Boards for Besins Healthcare and has received institutional research funding from Lawley Pharmaceuticals and provision of drug and placebo from Lawley Pharmaceuticals for clinical trials.
