# Supplementary material for: Testosterone and pre-androgens by age and menopausal stage at midlife: findings from a cross-sectional study
Source: eBioMedicine. 2025 Oct 15;121:105972. doi: 10.1016/j.ebiom.2025.105972 (PMC12552971; doi:10.1016/j.ebiom.2025.105972)
Supplement: Appendix [file mmc1.docx]

**Supplementary information S1**

Sex steroid measurement

Sex steroids were measured in a single serum sample by LC-MS/MS at the ANZAC Research Institute, University of Sydney, Australia as described previously,^6^ with the following modifications.

The ultrapressure liquid chromatography (LC) conditions included elution of steroids using a methanol/0.2mM ammonium fluoride in water gradient from a Kinetex XB C18 column (50 mm x 2.1 mm x 1.7 µm; part no. 00B-4498-AN) with a Phenomenex guard cartridge (UHPLC C18 for 2.1-mm ID columns, part no. AJ0-8782, Security Guard ULTRA Cartridges; Phenomenex, Lane Cove, New South Wales, Australia) at a column temperature of 45°C and flow rate of 0.35 mL/min. Chromatography running conditions provided baseline separation for each steroid, with a total run time of 7 minutes. The LC run was split into two periods to match the ionization requirements of the steroids. The steroids run in the second period (positive ionization mode) included testosterone (3.49 minutes), DHEA (3.36 minutes), and androstenedione (3.12 minutes).

Mass spectrometry was performed using an API-6500+ triple-quadrupole mass spectrometer (SCIEX, Foster City Concord, ON, Canada) equipped with an Electrospray Ionization (ESI) source that operated in both positive and negative ion modes (probe settings, X = 5, Y = 3). Nitrogen was used as curtain, nebulizer and collision gases. The curtain gas – -40psi, GS1 -70 psi, GS2 -60psi, Temp 600deg, IS -4500V, CAD-9. Multiple reaction monitoring with qualifier and quantifier transitions was used to quantify the steroids. The settings for the various transitions were optimized by infusing pure standard material into the mass spectrometer. Unit mass resolution was set in both mass-resolving quadrupole Q1 and Q3. A dwell time of 200 milliseconds was used for the target analytes and their corresponding isotopically labeled (deuterated) IS had a dwell time of 100 milliseconds. The quantifier transitions and collision energies (eV), respectively, were testosterone (289 to 109 and 35), androstenedione (287 to 97 and 28), and DHEA (271 to 213 and 28).

**Supplementary Table S1. Associations between body mass index, smoking and sex steroid concentrations**

|  | Univariable analysis | | Multivariable analysis* | |
| --- | --- | --- | --- | --- |
|  | β-coefficient (95% CI) | p | β-coefficient (95% CI) | p |
| **Testosterone (nmol/L)** |  |  |  |  |
| Body mass index, kg/m^2^ | 0.006 (0.001, 0.011) | 0.02 | 0.007 (0.002, 0.012) | 0.01 |
| Smoking (current vs never/past) | 0.116 (-0.014, 0.247) | 0.08 | 0.134 (0.002, 0.266) | 0.046 |
| **Dehydroepiandrosterone (nmol/L)** |  |  |  |  |
| Body mass index, kg/m^2^ | -0.006 (-0.010, -0.002) | 0.003 | -0.006 (-0.010, -0.002) | 0.003 |
| Smoking (current vs never/past) | 0.068 (-0.028, 0.165) | 0.17 | 0.042 (-0.052, 0.136) | 0.38 |
| **Androstenedione (nmol/L)** |  |  |  |  |
| Body mass index, kg/m^2^ | -0.0003 (-0.005, 0.004) | 0.91 | 0.001 (-0.003, 0.005) | 0.51 |
| Smoking (current vs never/past) | 0.237 (0.123, 0.350) | <0.001 | 0.218 (0.117, 0.318) | <0.001 |

*Generalized linear model of each sex steroid included age, body mass index, and smoking

**Supplementary Table S2. Sex hormone binding globulin (SHBG) concentrations by age***

|  | Age group (years) | | | | | |
| --- | --- | --- | --- | --- | --- | --- |
|  | 40-44  N=121 | 45-49  N=144 | 50-54  N=157 | 55-59  N=178 | 60-64  N=217 | 65-69  N=212 |
| **SHBG (nmol/L)** |  |  |  |  |  |  |
| Mean | 59.11 | 64.05 | 58.59 | 56.73 | 57.39 | 57.75 |
| Median | 54 | 60 | 54 | 51 | 53 | 49 |
| Standard deviation | 26.92 | 31.61 | 29.39 | 28.90 | 29.09 | 29.81 |
| Minimum | 14 | 11 | 10 | 10 | 9 | 14 |
| 10^th^ percentile | 31 | 26 | 26 | 25 | 23 | 27 |
| 90^th^ percentile | 100 | 103 | 97 | 98 | 94 | 106 |
| Maximum | 159 | 180 | 165 | 148 | 157 | 176 |

*No significant difference between any age group identified; 63 participants who had SHBG measured using a different platform ( Siemens Immulite 2000 XPi assay are excluded) Their inclusion/exclusion made no difference to the findings.

**Supplementary Table S3Associations between age and sex steroid concentrations adjusted for body mass index and smoking**

|  | Linear regression  β-coefficient (95% CI), p for age, r^2^ | Linear regression including (age)^2^  β-coefficient (95% CI), p for (age)^2^, r^2^ |
| --- | --- | --- |
| Testosterone (log-transformation) | -0.005 (-0.009, -0.001), p=0.007, 0.018 | 0.001 (0.0003, 0.001), p=0.003, 0.026 |
| Dehydroepiandrosterone (log-transformation) | -0.019 (-0.022, -0.015), p<0.001, 0.104 | -0.0004 (-0.001, -0.00001), p=0.055, 0.107 |
| Androstenedione (log-transformation) | -0.027 (-0.031, -0.024), p<0.001, 0.204 | 0.001 (0.001, 0.002), p<0.001, 0.226 |

**Supplementary Table S4. Sex steroid concentrations by menopausal stage in women aged 48-53 years**

|  | Median (IQR) |
| --- | --- |
| **STRAW+10** |  |
| **Testosterone (nmol/L)** |  |
| Premenopausal (n=63) | 0.53 (0.34, 0.86) |
| Perimenopausal (n=71) | 0.47 (0.35, 0.69) |
| Postmenopausal (n=58) | 0.51 (0.36, 0.67) |
| **Dehydroepiandrosterone (nmol/L)** |  |
| Premenopausal | 7.42 (5.65, 9.15) |
| Perimenopausal | 6.83 (5.17, 9.12) |
| Postmenopausal | 7.49 (5.10, 9.78) |
| **Androstenedione (nmol/L)** |  |
| Premenopausal | 1.94 (1.42, 2.54)^a^ |
| Perimenopausal | 1.71 (1.19, 2.25) |
| Postmenopausal | 1.63 (1.01, 2.02)^a^ |
| **Modified STRAW+10** |  |
| **Testosterone (nmol/L)** |  |
| Premenopausal (n=63) | 0.51 (0.34, 0.84) |
| Perimenopausal (n=71) | 0.48 (0.36, 0.69) |
| Postmenopausal (n=58) | 0.51 (0.36, 0.67) |
| **Dehydroepiandrosterone (nmol/L)** |  |
| Premenopausal | 7.38 (5.62, 9.15) |
| Perimenopausal | 7.07 (5.34, 9.12) |
| Postmenopausal | 7.49 (5.10, 9.78) |
| **Androstenedione (nmol/L)** |  |
| Premenopausal | 1.92 (1.42, 2.48)^b^ |
| Perimenopausal | 1.71 (1.21, 2.24) |
| Postmenopausal | 1.63 (1.01, 2.02)^b^ |

^a^p=0.001, ^b^p=0.007, between-group difference adjusted for age, body mass index, and smoking with Bonferroni correction

IQR: interquartile range

**Supplementary Table S5. Sex steroid concentrations by bilateral oophorectomy in postmenopausal women**

|  | Bilateral oophorectomy Yes n=60 | Bilateral oophorectomy No n=739 |
| --- | --- | --- |
| **Testosterone (nmol/L)** |  |  |
| Median (IQR) | 0.33 (0.25, 0.49)^a^ | 0.45 (0.32, 0.66)^a^ |
| **Dehydroepiandrosterone (nmol/L)** |  |  |
| Median (IQR) | 5.95 (4.44, 8.41) | 5.82 (3.99, 7.80) |
| **Androstenedione (nmol/L)** |  |  |
| Median (IQR) | 1.17 (0.79, 1.57) | 1.16 (0.87, 1.68) |

^a^p<0.001, between-group difference adjusted for age, body mass index, and smoking

IQR: interquartile range
